# Association Between Smoking Habits and Body Weight Among General Population in Saudi Arabia

**DOI:** 10.7759/cureus.51485

**Published:** 2024-01-01

**Authors:** Maryam Dahlawi, Muhammad Aldabbagh, Basant A Alzubaidy, Saif Dahlawi, Reema N Alotaibi, Wasan K Alsharif, Shahad B Alosaimi, Abdurahman Hassan-Hussein

**Affiliations:** 1 Medicine, Umm Al-Qura University, Makkah, SAU; 2 Community Medicine, Umm Al-Qura University, Makkah, SAU

**Keywords:** electronic cigarettes, smoking, bmi, body weight, obesity

## Abstract

Background: Smoking is a significant cause of mortality and is strongly associated with the development of morbid diseases, such as obesity. There is a considerable interest in understanding the impact of smoking on body weight. The exact nature remains unclear due to the mixed results observed in the literature review. The aim of this study is to evaluate the association between smoking and body weight concerning demographic characteristics in the western region of Saudi Arabia.

Methods: This cross-sectional study was carried out in the western region of Saudi Arabia from November 15, 2022 to July 15, 2023. To collect data, an online survey was utilized, and the survey link was shared across various social media platforms. The survey was completed by a total of 744 individuals who were at least 18 years old and answered a self-reported questionnaire on the web.

Results: Analysis showed that (39%) of participants lead a sedentary lifestyle, and (58.8%) have an abnormal BMI. Among them approximately 25% are overweight, 12.4% are obese, and 7.4% are extremely obese. Moreover, a greater number of them were traditional smokers. Our study reported that individuals who had quit smoking were more likely to be overweight. Regarding self-perception of their weight, almost half of the participants consider themselves overweight and they’re more likely to smoke, whether it be traditional or electronic cigarettes.

Conclusion: This study revealed a strong link between smoking habits and increased weight status, as measured by body mass indexes. These findings have important implications for public health interventions aimed at reducing smoking rates and obesity levels. By recognizing the potential connection between these two risk factors, experts in public health can develop more effective strategies to promote healthy behaviors and prevent chronic diseases among young individuals.

## Introduction

Smoking is a significant cause of mortality and is strongly associated with the development of severe illnesses that appear during adulthood [1]. Recent studies indicate that around 207,000 children aged 11 to 15 start smoking every year in the United Kingdom (UK), and the average age of smoking initiation is 13 in the United States (US). Furthermore, approximately 80% and 88% of adult smokers start smoking before the age of 20, respectively [2,3]. Early initiation of smoking is more likely to progress to regular smoking. Therefore, adolescence is a pivotal time to deliver smoking prevention programs for smoking uptake [3]. There is a considerable interest in understanding the impact of smoking on body weight. The exact nature remains unclear due to the mixed results observed in the literature review. The direction and magnitude of these effects varied across studies. Several studies have reported an association between smoking and lower weight and body mass index (BMI) [4]. However, there are also studies that suggest a contradictory effect of smoking, leading to a significant increase in BMI [5,6].

Although the connection between body weight and smoking among adolescents is intricate and not yet fully understood [3], various factors may contribute to their association. One of the most crucial factors is body dissatisfaction, which may act as a confounding or mediating variable in the relationship between BMI and smoking. High BMI may lead to body dissatisfaction, which, in turn, may result in increased smoking behavior [2]. Moreover, there is some evidence that high BMI is a possible risk factor for smoking initiation because people may start smoking in order to control or lose weight. 

The perception of one's own weight is another significant risk factor that appears to influence smoking initiation among adolescents. Adolescents' physical self-perception is a powerful influence on weight control behaviors, and a considerable proportion of them believe that smoking can help control body weight [7,8]. Studies have shown that adolescents who reported attempting to lose weight had higher rates of smoking initiation [9]. However, in another study, adolescent female smokers were not found to be more likely to be trying to lose weight than non-smokers. Biologically, smoking is generally perceived to decrease body weight due to factors such as decreased appetite and calorie intake, enhanced metabolism, and reduced fat accumulation. This may be attributed to the effects of nicotine on the brain's regulation of appetite and energy expenditure [10,11]. However, smoking decreases exercise by constraining respiratory functions. Smoking counteracts the previously mentioned effects on appetite and metabolism and results in increased body weight. Therefore, the biologic pathways suggest an ambiguous net effect of smoking on body weight [12]. There is still more to understand about the impact of smoking on body weight and unravel the exact nature of it as it is still unclear due to variation between studies.

The primary objective of this study is to evaluate the association between smoking and body weight concerning demographic characteristics. Additionally, we aim to investigate whether the perception of being overweight and body dissatisfaction contribute to smoking behavior among adolescents in the western region of Saudi Arabia. Unlike previous research, the unique focus of this study is placing a particular emphasis on the intensity of smoking, rather than just smoking initiation.

## Materials and methods

Study design

This cross-sectional study employed a web-based survey methodology, which was administered in the western region of Saudi Arabia, from November 15, 2022 to July 15, 2023. The online survey approach allowed for efficient data collection and facilitated widespread participation among the target population. The study was conducted by the Faculty of Medicine at Umm Al-Qura University, Makkah, Saudi Arabia.

Sampling strategy

The sample size was calculated by the Raosoft calculator for designing clinical research based on population size in the Western region (approximately 8,803,545); with obtaining 5% as a margin of error, 5% as a relative precision, and 95% as a confidence interval, the estimated sample was 385 participants. Individuals who were at least 18 years old and part of the general population residing in the western region of Saudi Arabia were eligible for participation. We used a convenience sampling technique by distributing an online survey link through various social media platforms including WhatsApp, Twitter, Telegram, and Facebook. Prior to commencing the survey, written consent was obtained from all participants through an initial page where they were required to provide their consent. The survey did not request any identifiable information from participants, those who provided answers outside our predefined criteria were excluded from the study, and only authorized research staff were granted access to the responses of the participants, thereby ensuring their privacy and confidentiality.

Study tool

A total of 744 individuals participated in the study, completing a web-based self-reported questionnaire that was adapted from two previously published studies [3,13]. The questionnaire consisted of four sections: (i) consent to participate, (ii) demographic information, (iii) data related to body weight, BMI, physical activity, and body perception, and (iv) information regarding smoking habits. The questionnaire was designed using Google Forms and data collection took place over one month. To ensure the security of the data, access was restricted to authorized personnel only. 

Ethical consideration

Approval from the Institutional Review Board (IRB: HAPO-02-K-012-2022-11-1244) at the College of Medicine, Umm Al-Qura University in Makkah, Saudi Arabia, was obtained on November 11.

Statistical analysis

The obtained data were initially gathered in an Excel sheet to be checked. Afterward, we used IBM SPSS for Windows version 23.0 (IBM Corp., Armonk, NY, USA) for the data analysis. The categorical data were presented as frequencies and percentages and the numerical data as means and standard deviations. The chi-square test was used to compare the categorical variables, and a p-value of ≤0.05 was considered to be statistically significant.

## Results

**Figure 1:**
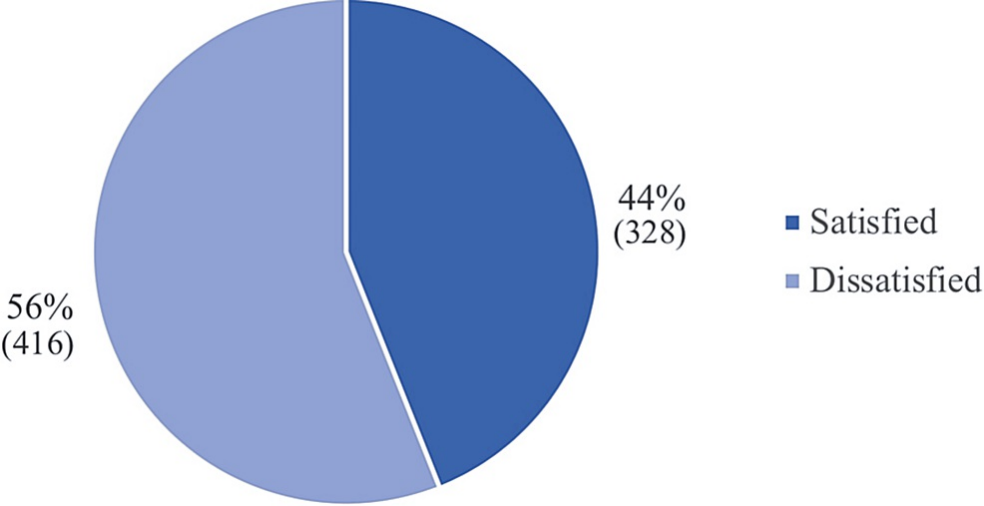
Participants satisfaction with their body weight

**Figure 2:**
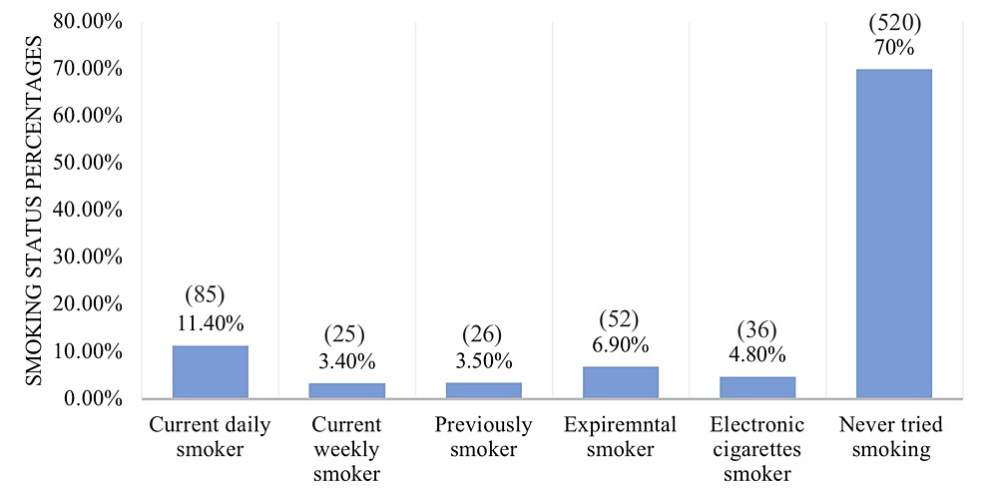
Smoking characteristics of participants

In this study, 744 individuals were included, and Table 1 presents an overview of their demographic characteristics. The largest proportion of participants (31.3%) falls within the age group of 21-25 years, and the majority (70.2%) are female. More than half of the participants are both bachelor's graduates (57.5%) and single (57.8%). In addition, 44.2% of the participants were students, and 33.2% were employed, with the majority of participants (54.6%) reporting a monthly income of less than 5,000 SR. Table 2 displays the body weight and physical activity traits of the participants. The majority of the participants (39%) lead a sedentary lifestyle, and a significant proportion (58.8%) have an abnormal BMI. Further, among those with abnormal BMI, approximately a quarter of the sample is overweight, 14% are underweight, 12.4% are obese, and 7.4% are extremely obese. In terms of self-perception of their weight, almost half of the participants consider themselves slightly overweight. According to Figures 1, 2, more than half of the participants in the sample expressed dissatisfaction with their body weight. Moreover, around (30%) of the participants had a positive smoking status, which can be further classified into 11.40% who are current daily smokers, 6.90% who have experimented with smoking, 3.50% who have quit smoking, 3.40% who are current weekly smokers, and 4.80% who are electronic cigarette smokers. Table 3 provides further detailed information regarding the smoking status of the participants.

**Table 1:** Demographics characteristics of participants SR: Saudi Riyals

Variables	Frequency	Percentage
Age		
18-20 years	152	20.4
21-25 years	233	31.3
26-30 years	69	9.3
31-35 years	57	7.7
36-40 years	45	6
41-45 years	49	6.6
46-50 years	53	7.1
51-55 years	38	5.1
More than 55 years	48	6.5
Gender		
Male	222	29.8
Female	522	70.2
Education		
Less than high school	40	5.4
High school	144	19.4
Diploma	71	9.5
Bachelor’s degree	428	57.5
Postgraduate degree (Masters, PhD)	61	8.2
Marital status		
Married	294	39.5
Single	430	57.8
Widow	10	1.3
Divorced	10	1.3
Total perceived family income per month		
Less than 5000 SR	406	54.6
5000-10000 SR	143	19.2
More than 10000	195	26.2
Occupation		
Employed	247	33.2
Unemployed	106	14.2
Student	329	44.2
Retired	62	8.3

**Table 2:** Body weight and activity characteristics of participants BMI: Body Mass Index

Variables	Frequency	Percentage
Physical activity		
Sedentary lifestyle (<5000 steps per day)	290	39
Low active (5000-7499 steps per day)	199	26.7
Somewhat active (7500-9999 steps per day)	183	24.6
Active (>10000 steps per day)	54	7.3
Highly active (>12500 steps per day)	18	2.4
BMI		
Underweight <18.5	104	14
Normal 18.5-24.9	307	41.3
Overweight 25-29.9	186	25
Obese 30-34.9	92	12.4
Extremely obese >35	55	7.4
Perception of weight		
Underweight	107	14.4
About the right weight	221	29.7
Slightly overweight	332	44.6
Extremely overweight	82	11.3

**Table 3:** Frequency and duration of smoking for participants

Variables	Frequency	Percentage
Current daily traditional smoker		
Duration		
1-3 years	20	23.5
4-6 years	14	16.5
6-10 years	19	22.4
More than 10 years	32	37.6
Number of cigarettes per day		
Less than 5 cigarettes per day	15	17.6
5-10 cigarettes per day	40	47.1
11-20 cigarettes per day	22	25.9
More than 20 cigarettes per day	8	9.4
Current weekly traditional smoker		
Duration		
1-3 years	13	52
4-10 years	11	44
More than 10 years	1	4
Number of days per week		
1-2 days	17	68
3-4 days	8	32
Number of cigarettes per week		
Less than 5 cigarettes per week	21	84
5-10 cigarettes per week	3	12
11-20 cigarettes per week	1	4
Previously smoker		
Duration of smoking cessation		
Less than 1 year	13	50
1-3 years	1	3.8
4-6 years	1	3.8
7-10 years	6	23.1
More than 10 years	5	19.2
Electronic cigarettes smoker		
Duration		
1-3 years	31	86.1
4-6 years	1	2.8
6-10 years	2	5.6
More than 10 years	2	5.6

Table 4 reports the relationship between smoking habits and body weight. Our analysis revealed that individuals under the age of 45 were more likely to smoke. Moreover, there was a significant correlation between male gender and traditional smoking, while females were more inclined towards experimental smoking and electronic cigarettes, with a statistically significant p-value (P=0.000). In terms of education, traditional smoking was prevalent among participants with a bachelor's or diploma degree, while electronic cigarettes were commonly used among those with a bachelor's or high school certification. We observed a statistically significant p-value (p=0.011) indicating that traditional smoking was more prevalent among married participants, whereas electronic cigarettes were more commonly used by single individuals. Additionally, our study revealed a significant correlation (p=0.000) between smoking prevalence and perceived income, with a higher prevalence of smoking observed among those with lower perceived income. Furthermore, we found that traditional smoking was more common among employed participants, whereas electronic cigarettes were more popular among student participants. In regard to the relationship between smoking and BMI, our study found that a greater number of overweight, obese, and extremely obese participants were traditional smokers. Additionally, individuals who had quit smoking were more likely to be overweight. We also observed a significant correlation between participants' perception of their weight and smoking habits, with those who considered themselves to be overweight more likely to smoke, whether it be traditional or electronic cigarettes. The p-values were generated using the chi-square test in SPSS software, providing statistical evidence to assess the significance of the observed associations in the study.

**Table 4:** Association between smoking, demographics factors, and body weight BMI: Body Mass Index, SR: Saudi Riyal

Variables	Traditional smoker	Previously smoker	Experimental smoker	Electronic cigarettes smoker	Never tried smoking	P-value
Age						
18-20 years	11	1	4	6	130	0.001
21-25 years	30	6	20	12	165	
26-30 years	17	2	7	4	39	
31-35 years	10	5	6	4	32	
36-40 years	10	2	6	2	25	
41-45 years	14	4	3	4	24	
46-50 years	8	3	2	1	39	
51-55 years	4	2	2	0	30	
More than 55 years	6	1	1	3	37	
Gender						
Male	76	22	20	17	87	0.000
Female	34	4	31	19	434	
Education						
Less than high school	12	0	0	1	27	0.000
High school	20	6	9	11	98	
Diploma	25	1	4	1	40	
Bachelor’s degree	45	13	30	19	321	
Postgraduate degree	8	6	8	4	35	
Marital status						
Married	58	18	21	18	179	0.011
Single	52	8	29	18	323	
Widow	0	0	0	0	10	
Divorced	0	0	1	0	9	
Income						
Less than 5000 SR	49	6	28	19	304	0.000
5000-10000 SR	30	7	4	5	93	
More than 10000	31	13	15	12	124	
Occupation						
Employed	55	19	20	14	139	0.000
Unemployed	21	2	8	4	71	
Student	28	5	21	16	259	
Retired	6	0	2	2	52	
BMI						
Underweight <18.5	13	2	5	3	81	0.034
Normal 18.5-24.9	41	5	24	17	220	
Overweight 25-29.9	33	12	13	10	118	
Obese 30-34.9	13	7	7	2	63	
Extremely obese >35	10	0	2	4	39	
Perception of weight						
Underweight	24	4	4	7	68	0.058
About the right weight	36	3	17	10	155	
Slightly overweight	38	15	24	15	240	
Extremely overweight	12	4	6	4	58	
Satisfaction about weight						
Satisfied	46	7	20	13	242	0.133
Dissatisfied	64	19	31	23	279	

## Discussion

Smoking and obesity are two major public health concerns that have gained widespread attention worldwide. Smoking is a leading cause of preventable deaths and is associated with several health risks [14]. On the other hand, obesity is a major risk factor for chronic diseases. The association between smoking and obesity has been a topic of interest in public health research for many years and several theories have been proposed to explain the potential link between smoking and obesity. Despite the numerous studies conducted on this topic, there is still a need for further understanding of the nature and extent of this association [15]. Thus, this research was conducted to investigate if there is a significant correlation between these two factors.

In the present study, which included 744 participants from the general population in the western region of Saudi Arabia, a substantial proportion (39%) reported a sedentary lifestyle. Additionally, an extensive proportion of the sample, amounting to 58.8%, had an abnormal BMI. Further analysis of the data revealed that among those with an abnormal BMI, around a quarter of the participants were overweight, 14% were underweight, 12.4% were obese, and 7.4% were extremely obese. In comparison to a study conducted in the US [3] that examined the link between body weight and smoking behavior, they revealed that 14.9% of their participants had an overweight BMI and 17.3% were obese, which is similar to the BMI distribution in our sample.

The actual BMI and the perception of weight can differ significantly. The perception of weight is a subjective experience that can be influenced by a range of factors, including cultural norms, media images, and personal experiences. People may perceive themselves as heavier or lighter than their actual BMI due to these factors, which can lead to body dissatisfaction. Our study explored how participants perceived their own weight, revealing that nearly half of them viewed themselves as slightly overweight. This finding contrasts with a previous study conducted in the US [3], which found that the majority of their participants (56.6%) believed they were at an appropriate weight, while only a minority (25.7%) considered themselves slightly overweight. Furthermore, over 50% of the participants included in our sample expressed dissatisfaction with their body weight. This finding aligns with the results of a previous study carried out in the UK [2].

In our study, we observed a higher proportion of overweight, obese, and extremely obese individuals among traditional daily or weekly smokers, providing evidence for a positive association between smoking habits and higher weight status, as indicated by the body mass index (BMI). This finding contrasts with a study conducted in Thailand [5], which demonstrated a negative association between smoking and BMI. Moreover, in the current study, we reported that individuals who had quit smoking were more likely to be overweight, this is similar to the findings of a study published in China [16] which revealed that quitting smoking is associated with an increased risk of becoming overweight or obese and that smoking cessation is moderately associated with weight gain. Two other studies also reported substantial weight gains associated with smoking cessation by comparing current and former smokers [17,18]. The researchers suggest that the increased risk of weight gain after quitting smoking may be attributed to alterations in metabolism and appetite regulation, as well as the psychological effects of smoking cessation. In addition, the fear of gaining weight could discourage current smokers from attempting to quit. Therefore, we suggest that more research should be done to explore interventions aimed at preventing or reducing post-cessation weight gain. In our study, a significant association was found between participants' perception of their weight and smoking habits, as those who considered themselves to be overweight more likely to smoke, whether it be traditional or electronic cigarettes. This is similar to the US study [3], which reported a significant relationship between the perception of being overweight and the increased likelihood of smoking among adolescents, especially among female adolescents.

Our study suggests that higher BMI and the perception of being overweight may be associated with an increased risk of subsequent smoking behavior among adolescents. Further research is needed to explore the relationship between BMI and smoking in other regions of Saudi Arabia. Addressing body weight perception and BMI during adolescence could be a crucial step in preventing the onset of smoking and could also serve as a starting point for smoking cessation campaigns by targeting and treating obesity, as managing obesity can be beneficial in preventing the complications associated with obesity itself, as well as reducing the likelihood of smoking initiation and preventing the dual complications and chronic diseases that can arise from smoking and obesity. Therefore, efforts to manage obesity could have a significant impact on public health by addressing both obesity and smoking-related health issues.

Moreover, our study revealed several interesting findings regarding smoking habits among participants. Firstly, traditional smoking was more prevalent among employed individuals and males, whereas experimental smoking and electronic cigarette use were more common among females. Secondly, married participants were more likely to engage in traditional smoking than their single counterparts, who were more likely to use electronic cigarettes. These findings have important implications for public health interventions aimed at reducing smoking rates, particularly in the Saudi Arabian context. For instance, targeting traditional smoking among employed individuals and married individuals could be an effective strategy for reducing smoking rates in these populations. Additionally, interventions designed to promote smoking cessation among males may be more effective if they specifically target traditional smoking, whereas interventions aimed at females may need to consider the popularity of electronic cigarettes among this group. Overall, these findings highlight the importance of considering gender, employment status, and marital status when designing smoking cessation interventions tailored to the unique needs of different populations.

It is crucial to acknowledge and address the limitations in this study. Firstly, the reliance on voluntary participation introduces the potential for selection bias, which may limit the generalizability of the findings to the entire population of Saudi Arabia. The data represent a specific region within Saudi Arabia, namely the western region. Therefore, caution should be exercised when extrapolating the results to the broader population. Secondly, the use of self-reported data collected through an online survey introduces the possibility of response bias. These limitations should be taken into account when interpreting the study's findings and drawing conclusions. To enhance the generalizability of the results, it is recommended to conduct further research encompassing various regions of Saudi Arabia. This would involve gathering data from more diverse and representative samples, allowing for a more comprehensive understanding of the topic in the whole Saudi Arabia.

## Conclusions

This research of the general population in the western region of Saudi Arabia, highlights a significant association between smoking habits and higher weight status, as evidenced by body mass indexes. These results carry crucial implications for public health interventions targeting the reduction of smoking prevalence and obesity rates. By recognizing the potential association between these two risk factors, public health experts can devise more impactful strategies to encourage healthy behaviors and mitigate the onset of chronic diseases among adolescents.
